# Holistic impact assessment and cost savings of rainwater harvesting at the watershed scale

**DOI:** 10.1525/elementa.135

**Published:** 2017

**Authors:** Santosh R. Ghimire, John M. Johnston

**Affiliations:** * Oak Ridge Institute for Science and Education (ORISE) Postdoctoral Research Participant U.S. Environmental Protection Agency, Office of Research and Development, Athens, Georgia, US; † U.S. Environmental Protection Agency, Office of Research and Development, Athens, Georgia, US

**Keywords:** watershed scale, rainwater harvesting, sustainability, life cycle cost and impact

## Abstract

We evaluated the impacts of domestic and agricultural rainwater harvesting (RWH) systems in three watersheds within the Albemarle-Pamlico river basin (southeastern U.S.) using life cycle assessment (LCA) and life cycle cost assessment. Life cycle impact assessment (LCIA) categories included energy demand, fossil fuel, metals, ozone depletion, global warming, acidification, smog, blue and green water use, ecotoxicity, eutrophication, and human health effects. Building upon previous LCAs of near-optimal domestic and agricultural RWH systems in the region, we scaled functional unit LCIA scores for adoption rates of 25%, 50%, 75%, and 100% and compared these to conventional municipal water and well water systems. In addition to investigating watershed-scale impacts of RWH adoption, which few studies have addressed, potential life cycle cost savings due to reduced cumulative energy demand were scaled in each watershed for a more comprehensive analysis. The importance of managing the holistic water balance, including blue water (surface/ground water), green water (rainwater) use, and annual precipitation and their relationship to RWH are also addressed. RWH contributes to water resource sustainability by offsetting surface and ground water consumption and by reducing environmental and human health impacts compared to conventional sources. A watershed-wide RWH adoption rate of 25% has a number of ecological and human health benefits including blue water use reduction ranging from 2–39 Mm^3^, cumulative energy savings of 12–210 TJ, and reduced global warming potential of 600–10,100 Mg CO_2_ eq. Potential maximum lifetime energy cost savings were estimated at $5M and $24M corresponding to domestic RWH in Greens Mill and agricultural RWH in Back Creek watersheds.

## Introduction

Regarding our global water footprint, Hoekstra and Mekonnen (2012) describe consumptive use of surface and groundwater as blue water, rainwater as green water, and waste assimilation water as gray water. Consumptive use can be direct use (including evaporated water) or indirect use (embedded volume of water in a product’s full supply chain); non-consumptive water use (return flow) is not included in any water footprint (WFN 2009). Worldwide from 1996–2005, rain-fed agricultural production had a 91% green and 9% gray water footprint, while irrigated agriculture had 48% green, 40% blue, and 12% gray water footprint (Hoekstra and Hung 2002; Chapagain and Hoekstra 2004; Mekonnen and Hoekstra 2011).

Rainwater harvesting (RWH) is a technique to capture and store green water for future use. Examples include rain barrels and cisterns for non-potable household use and farm ponds for crop irrigation and livestock watering. RWH is also a climate change adaptation strategy and rehabilitates ecosystems services for human well-being by reducing stormwater runoff, mitigating sewer overflows, conserving soil, and enhancing food and economic security (USEPA 2008c; UNEP-SEI 2009). By increasing the use of green water, RWH can augment conventional centralized water supplies during water shortages and may function as a decentralized water system for climate change adaptation (Sedlak 2014). Globally, 71% of irrigated areas and 47% of large cities are reported to experience periodic water shortages (Brauman et al. 2016). RWH practices widely used in Asia and Africa are of interest in the U.S., such as irrigation in North Carolina, Virginia, Texas, and Ohio. These states promoted RWH in their regulations as well (TWDB 2006; DeBusk et al. 2013; Shuster et al. 2013; ARCSA 2014; Thomas et al. 2014). In the southeastern U.S., RWH is getting attention due to recent droughts (DeBusk et al. 2013), and RWH adoption will likely increase in the face of unreliable rainfall during the growing season. While most of the Southeast received heavy downpours in recent autumns, it also observed increases in moderate-to-severe drought in spring and summer (12% and 14%) from 1970 to 2007 (USGCRP 2009; 2012; USEPA 2015).

“Decreased water availability is very likely to affect [the Southeast] region’s economy as well as its natural systems” (USGCRP 2012). This is due to climate impacts combined with increased water demand, such as a 20% increase in agricultural irrigation by surface and groundwater withdrawals in Georgia, anticipated to continue through 2050 (Hook et al. 2009). However, adoption of RWH to compensate for water needs can introduce environmental and human health impacts and their associated economic costs (USGCRP 2009; 2012). RWH impoundments can impact biodiversity and wetland and upland habitat by interrupting stream ecosystems (USEPA 1990). The number of small reservoirs in the Georgia Piedmont increased from 19 to 329, mostly due to agricultural practices from 1950–1970 and suburban growth from 1980–1990 (Ignatius and Jones 2014). Although water resource decisions are local, the scope of impact is national. Approximately 20% of 2.6 million small water bodies account for the majority of standing water areas across the conterminous U.S. (Smith et al. 2002), and approximately half of the estimated 80,000 ponds for fishing or irrigation in the Coastal Plain of Georgia are man-made (Vellidis 2015). Therefore, understanding the economic, environmental and human health impacts of RWH is crucial in decision making, especially in the context of current global climate change and water scarcity.

Life cycle impacts associated with production, installation and maintenance to point-of-use of domestic and agricultural RWH systems include greenhouse gas (GHG) generation, energy use, smog, hydrologic impacts on blue water, and human and ecological health impairment (Ghimire et al. 2014). Life cycle assessment (LCA) is a widely accepted decision-support framework that assesses environmental and human health impacts of systems/products in a cradle-to-grave approach. The International Organization for Standardization (ISO 2006b; 2006a) provides LCA’s framework which includes goal and scope definition, inventory analysis, and life cycle impact assessment (LCIA). LCIA methods like the Tool for the Reduction and Assessment of Chemical and Other Environmental Impacts, or TRACI (USEPA 2012), characterize environmental emissions utilizing inventory data such as that compiled and recommended by the U.S. Environmental Protection Agency (EPA), National Institute of Standards and Technology, and Department of Energy (NIST 2013a; NREL 2013; USEPA 2014b) in conjunction with commercially available databases. LCA thus addresses problem-shifting effects with a systematic, holistic approach that produces a comprehensive suite of environmental and human health impact indicators of sustainability. Prior LCA modeling of domestic RWH at household level and agricultural RWH at farm level found decreased environmental and human health impacts compared to conventional municipal drinking water and well water (Ghimire et al. 2014).

Previous studies have explored the feasibility and benefits of RWH. Hydrologic feasibility of watershed-wide adoption of RWH was modeled in three watersheds within the Albemarle-Pamlico river basin in North Carolina and Virginia, where a 25% adoption rate reduced downstream water yield 6%-16% (Ghimire and Johnston 2013). Other U.S. examples include economic, environmental, and water quality evaluation of RWH in the midwestern U.S. (Anand and Apul 2011; Ghimire et al. 2012; Shuster et al. 2013) and RWH performance in the southeastern U.S. (Jones and Hunt 2010; Steffen et al. 2013). In Switzerland, Crettaz et al. (1999) performed LCA of RWH for toilet flushing and recommended a combined system of conventional and low-flushing toilets for environmental benefits. An LCA of RWH for laundry use in Spain’s Mediterranean climate compared environmental impacts of various urban densities and reported favorable performance in compact density (Angrill et al. 2012). Globally, others have focused on RWH design in Taiwan (Liaw and Tsai 2004); RWH viability in Bangladesh and India (Alam et al. 2012; Van Meter et al. 2014); water saving, cost-effectiveness potential, and water quality evaluation of RWH in Sweden, Australia, and the UK (Villarreal and Dixon 2005; Eroksuz and Rahman 2010; Tam et al. 2010; Ward et al. 2012; van der Sterren et al. 2014); runoff volume reduction in China (Zhang and Hu 2014); hydrologic assessment in Africa and India (Ngigi et al. 2005; Glendenning and Vervoort 2010); RWH contamination issues (Lye 2009); and LCA environmental and cost impacts in Europe (Roebuck et al. 2011). Other countries exploring RWH include Singapore, Japan, Germany, Thailand, Indonesia, the Philippines, and Brazil (UN 2014). The economic feasibility, hydrologic feasibility, environmental impacts, energy and water savings of RWH varies with design, water demand and rainfall availability, geographic location (topography), and pumping energy needs. Moreover, an understanding of RWH impacts at wider adoption scales (such as watershed-scale) holistically is an unknown, which is the primary driver of this study.

A watershed-wide approach is preferred to address complex water resource and environmental management challenges including water supply and recreation demands, aquatic habitat protection, and water quality regulations, as well as consideration of RWH and other green infrastructure practices (USEPA 1996; 2008b). The USEPA’s watershed ecological risk assessment process guides problem formulation, analysis, and risk characterization to improve environmental decision making (Bruins and Heberling 2004; USEPA 2008a). The goal is to provide a more comprehensive perspective on watershed-wide impacts of RWH to support sustainable use of water resources and minimize unintended consequences such as creation of new problems. We focus in this study on domestic RWH for toilet flushing and agricultural RWH for crop irrigation.

### Objective and novelty

Our objective is to comprehensively address the impacts of RWH at the watershed scale, including LCIA impact categories of energy use, GHG emissions, human and ecological health, in addition to life cycle water balance and life cycle energy cost savings. The study builds upon previous work (Ghimire et al. 2014) that compared the LCIA of household-level domestic RWH with municipal drinking water for toilet-flushing and farm-level agricultural RWH with well water for crop irrigation. We analyze scaled, functional unit (normalized to 1 m^3^ water supply) impacts of cumulative energy demand, fossil fuel depletion, global warming potential, metal depletion, ozone depletion, acidification, smog, blue water use, green water use, ecotoxicity-total, eutrophication-total, human health criteria pollutants, human health cancer, and human health non-cancer. This study also addresses (1) the variation in domestic and agricultural RWH adoption rates; (2) differences in holistic blue water savings; and (3) differences in cumulative energy cost savings in two types of RWH practices. While gray water use is an important consideration, it was not included due to LCA data limitations.

Net water balance between reduced life cycle blue water (surface and ground water) use and harvested rainwater (green water) use, watershed-wide is also of interest. We evaluated net blue water savings by incorporating the loss in annual water yield due to watershed-wide RWH. The loss in water yield incorporates rainfall influences combining surface runoff, lateral flow, groundwater contribution (return flow from shallow aquifer), transmission losses, and pond abstractions. The difference in potential cost savings from the life cycle’s cumulative energy demand reduction are addressed for each watershed by performing life cycle cost assessment (LCCA) of an agricultural RWH system, contrasting it with the capital costs of RWH installation.

Our approach utilizes functional unit LCIA impacts and embraces systems thinking through incorporation of environmental, economic, and social dimensions of sustainability relevant to other locations. To our knowledge, no previous study has simultaneously addressed watershed-wide environmental and human health impacts and potential energy and cost savings of RWH.

## Methods

**Figure 1:**
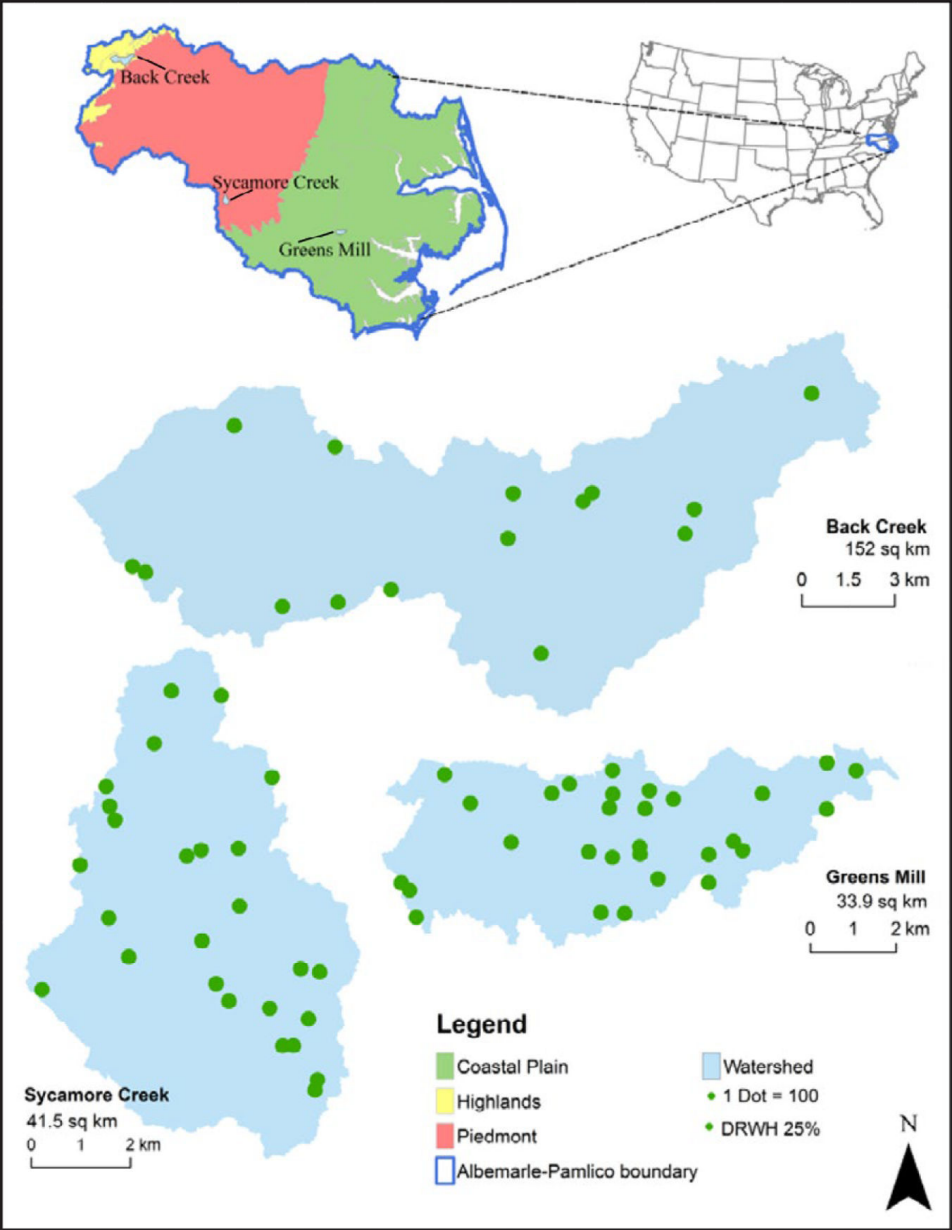
Study area within the Albemarle-Pamlico basin with 25% adoption rate for domestic RWH (DRWH) systems (1 dot = 100). Figure modified from Ghimire and Johnston (2013). DOI: https://doi.org/10.1525/elementa.135.f1

The study area includes Back Creek (152 km^2^ Hydrologic Unit Code or HUC # 30101010405), Sycamore Creek (41.5 km^2^ HUC # 30202010802), and Greens Mill Run (33.9 km^2^ HUC # 30201030403) within the Albemarle-Pamlico river basin in North Carolina and Virginia (Figure 1). These watersheds span a rural to urban land use gradient from the Highlands of Virginia to the Coastal Plain of North Carolina and include a total of 5,768, 10,296, and 11,582 households, and 34, two, and three farms, respectively (Table 1). The 30-year (1980–2009) mean annual precipitation is 106 cm for Back Creek, 111 cm for Sycamore Creek, and 127 cm for Greens Mill Run (Ghimire and Johnston 2013). RWH includes domestic systems for toilet-flushing equal to the number of households and agricultural systems for corn irrigation equal to the number of farms in each watershed consistent with near-optimal RWH designs from Ghimire et al. (2014). The near-optimal RWH systems minimize infrastructure through shortened pipe lengths and maximize the use of gravity for RWH delivery.

We used adoption rates of 25%, 50%, 75% and 100%, with 25% a reasonable lower rate in light of increased interest in RWH to span the potential range of adoption, consistent with prior studies (Kahinda et al. 2009; Ghimire and Johnston 2013). Thomas *et al.* (2014) reported that approximately 90% of respondents used harvested rainwater for irrigation, and more than 25% of the harvested water was used for potable purposes. We also consulted water resource management researchers from various organizations regarding the potential for RWH adoption in the region, including The University of Georgia, EPA Region IV, the Natural Resources Conservation Service, the U.S. Army Corps of Engineers, and the Southeast representative of the American Rainwater Catchment Systems Association.

Total household and farm numbers reflect the actual state of the watersheds. RWH systems are not currently installed and represent potential adoption scenarios. We estimated the number of agricultural RWH systems (*N*_*t*_) in each watershed based on average area of a family farm (*a*) and actual area (*A*_*T*_); see Equation 1 and Table 1 (Ghimire and Johnston 2013).

(1) $$N_{t}=\frac{A_{T}}{a}$$

**Figure 2:**
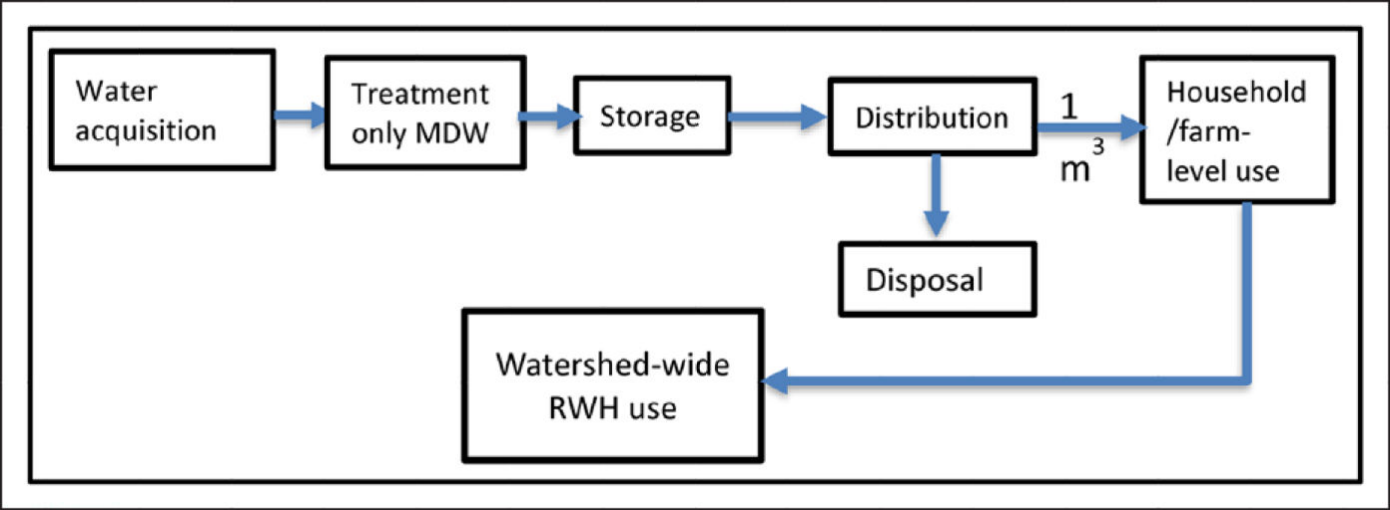
LCA system boundary for domestic rainwater harvesting (RWH), agricultural RWH, municipal drinking water (MDW), and well water from household/farm-level to watershed wide use (adapted from Ghimire et al. 2014): Water acquisition refers to RWH, surface water for MDW, and well water for irrigation. DOI: https://doi.org/10.1525/elementa.135.f2

Different adoption scenarios represent scaling of RWH at the specified percentage. 100% agricultural RWH adoption equates to the ratio of all available farm areas to the designated average family farm size defined by Equation 1. 50% adoption represents half of total available farm area, 0.5 × *N*_*t*_. Agricultural RWH adoption represented the whole or partial farm, depending on percentage of adoption by all farms. For example, 25% adoption of total agricultural RWH systems in Sycamore Creek means that farmers utilized agricultural RWH in 25% of the available farm area. We estimated the number of domestic RWH systems equal to the household units in each watershed, obtained from the U.S. Census Bureau’s Topologically Integrated Geographic Encoding and Referencing housing units database (Table 1) (Bureau 2011). The LCA system boundary for domestic RWH and agricultural RWH is shown in Figure 2.

### Watershed-wide LCA impact calculations

Change or reduction in each impact category of domestic RWH and agricultural RWH was calculated with respect to baselines of conventional municipal drinking water and well water irrigation. Equation (2) quantified the change in watershed-scale life cycle impacts, based on functional unit impacts of 1 m^3^ of rainwater delivery with respect to a conventional water supply:

(2) $$I_{w}=A\times N_{t}\times Q_{y}\times T\times \Delta i$$

where

*I*_*w*_ = change in watershed scale impact (Units)

*A =* RWH adoption rates (0.25, 0.50, 0.75, and 1)

*N*_*t*_ *=* total number of RWH systems (Table 1)

*Q*_*y*_ *=* annual water demand for household toilet flushing or corn crop irrigation (m^3^/y): the annual household toilet flushing water demand was estimated at 37 m^3^/y using average demand at 37.8 liter per capita per day for low-flush toilet (Ghimire et al. 2014) and crop irrigation demand estimated at 90,900 m^3^/y using 606 m^3^/day for 150 days per year irrigation (Ghimire and Johnston 2013)

*T =* service life of a RWH system (50 y)

*Δi* = *i*_*con*_ − *i*_*rwh*_ = difference in impact per 1 m^3^ of rain-water delivery, *i*_*rwh*_, with respect to conventional water supply, *i*_*con*_, (Units/m^3^), where Units are as follows: cumulative energy demand (MJ), fossil fuel depletion (kg oil eq), global warming potential (kg CO_2_ eq), metal depletion (kg Fe eq), ozone depletion (kg CFC11 eq), acidification (kg SO_2_ eq), smog (kg O_3_ eq), blue water use (m^3^), green water use (m^3^), ecotoxicity-total (CTU or comparative toxic units), eutrophication-total (kg N eq), human health criteria pollutants (kg PM_2.5_ eq), human health cancer (CTU), and human health non-cancer (CTU).

### Life cycle assessment (LCA) methods

A prior study (Ghimire et al. 2014) briefly summarized here provided the functional unit LCIA impacts (per m^3^ water delivery) of domestic RWH at household level, agricultural RWH at farm level, conventional municipal drinking water for toilet flushing, and well water for irrigation. Calculations of impact per functional unit (i.e., *i*_*con*_ and *i*_*rwh*_, in Equation 1) were performed using OpenLCA (OpenLCA 2013), a publicly available software linked to the Ecoinvent database and the EPA’s LCIA methods. LCIA methods incorporated blue and green water use from water footprint methods (WFN 2009), fossil and metal depletion methods from ReCIPE (Goedkoop et al. 2012), and the non-renewable cumulative energy demand method from Ecoinvent version 2.2 (Hischier et al. 2010) within TRACI version 2.0 (USEPA 2013). TRACI’s characterization of human toxicity and ecotoxicity was adapted from the USETox Model and adjusted to remove characterization of metal toxicity due to uncertainty (Bare 2002; Hauschild et al. 2008; Ghimire et al. 2014).

Details on each impact category in TRACI are available from Bare (2011) and the USEPA (2012). TRACI utilizes the amount of chemical emission or resource used to estimate potential impact of each category, as described by Equation 3:

(3) $$I_{j}=\sum_{km}CF_{km.j}\times M_{km}$$

where,

for a specific impact category, *j*:

*I*_*j*_ = the potential impact of all chemicals (k)

*CF*_*km.j*_ = the characterization factor of chemical (k) emitted to media (m)

*M*_*km*_ = the mass of chemical (k) emitted to media (m)

Our method (Equation 2) is based on linear scaling of the functional unit impacts compared to conventional water supplies. It should be noted that virtually all LCA studies (and databases) utilize simplified models of linear form, and normalizing unit process flows to functional unit is a widely accepted LCA practice (Heijungs and Suh 2002). Heijungs and Suh (2002) considered this concept mathematically by describing the scaling vector *s* as a function of external demand (output flow) vector *d* and the inverse of flow matrix *T* (flows) in estimating unit process in a system, or *s* = *T*^−1^*d.* Economies of scale arising from wider adoption of RWH systems are beyond the scope of this study.

### Life cycle inventory

Life cycle inventory (LCI) details including data inventory sources, design parameters, and underlying assumptions were described in a prior study (Ghimire et al. 2014), with near-optimal RWH system designs minimizing infrastructure through shortened pipe lengths and maximizing gravity for delivery. The near-optimal domestic RWH systems used a polyethylene storage tank of 6.2 m^3^, reduced pipe length (5 m) of chlorinated polyvinyl chloride, and eliminated the pump and pumping energy. Similarly, the near-optimal agricultural RWH used polyvinyl chloride pipe of 150 m, polyethylene storage tank of 606 m^3^, and no pump or pumping energy. The Building for Environmental and Economic Sustainability database (NIST 2013a) and a European database (Ecoinvent 2012) were used to complete the materials inventory.

### Life cycle water balance

We evaluated net life cycle blue water (surface and ground water) savings by incorporating the loss in annual water yield due to watershed-wide RWH. The loss in water yield incorporates rainfall influences combining surface runoff, lateral flow, groundwater contribution (return flow from shallow aquifer), transmission losses, and pond abstractions. Net water balance between reduction in life cycle blue water use and green water use is described (Equation 4):

(4) $$W_{b}=BU_{r}-GU$$

where

*W*_*b*_ = life cycle water balance in a watershed (Mm^3^)

*BU*_*r*_ = net reduction in life cycle blue water use (Mm^3^)

*GU* = life cycle green water use (i.e., harvested rainwater) (Mm^3^)

Life cycle water balance, *W*_*b*_, is estimated utilizing the watershed scale savings in blue water and green water obtained from Equation 2. Equation 4 estimates reduction in embedded blue water in all life cycle stages of RWH compared to green water harvesting over a 50 year lifetime. Blue water consumption or withdrawal does not include release to the same water body. Equation 4 also does not include rainfall influences. We integrate rainfall influence on life cycle water balance using watershed annual water yield loss, i.e., the combination of surface runoff, lateral flow, groundwater contribution, transmission losses, and pond abstractions in each watershed for 100% domestic and agricultural RWH adoption (Equation 5):

(5) $$B_{H}=BU_{y}-W_{a}\times R_{y}$$

where,

*B*_*h*_ = annual blue water savings due to domestic or agricultural RWH, compared to conventional water supplies, by integrating rainfall (m^3^/y)

*BU*_*y*_ = annual net savings in life cycle blue water use due to domestic or agricultural RWH, compared to conventional water supplies (m^3^/y) (see Tables 2 and 3)

*W*_*a*_ = watershed area contributing to total water yield (m^2^) (see Table 1)

*R*_*y*_*=* the loss in annual water yield due to domestic or agricultural RWH, compared to no-RWH annual water yield (m^3^/y) (Equation 6):

(6) $$R_{y}=\sum_{i=1}^{12}{\text{Y}}_{i.0}-\sum_{i=1}^{12}{\text{Y}}_{i.R}$$

where,

*Y*_*i.o*_ is the monthly water yield for No-RWH and *Y*_*i.R*_ is the monthly water yield for the specified RWH adoption within the watershed (m/month). Water yield, *Y*_*i*_ for each RWH system, is defined as the net amount of water leaving the watershed during the monthly time step (Equation 7) obtained from Ghimire and Johnston (2013):

(7) $$Y_{i}=\left(\frac{1}{1000}\right)\times (S+L+G-R-P)$$

where, for monthly time step,

*Y*_*i*_ = net amount of water leaving the watershed during the monthly time step (m/month)

*S* = surface runoff contribution to streamflow (mm/month)

*L* = lateral flow (within the soil profile) contribution to streamflow (mm/month)

*G* = groundwater contribution (return flow from shallow aquifer) to streamflow (mm/month)

*R* = transmission losses (becomes recharge for the shallow aquifer) from tributary channels via transmission through the bed (mm/month)

*P* = pond abstractions (net change in water volume of pond) (mm/month)

$\left(\frac{1}{1000}\right)$ = a conversion factor, i.e., $\left(\frac{1}{1000}\right)m=1mm$

*Y*_*i*_*s* (Equation 6) were obtained from Ghimire and Johnston (2013) who used the Soil and Water Assessment Tool (SWAT) model developed by the U.S. Department of Agriculture to simulate change in water yield (*Y*_*i*_s) of domestic and agricultural RWH systems within the three watersheds (Arnold et al. 2011). Thirty-year (1980–2009) daily rainfall data (NCDC 2012) were used as input, and for details on SWAT-based RWH model development, calibration and validation, see Ghimire and Johnston (2013).

### Life cycle cost savings due to cumulative energy demand reduction

Energy cost savings from watershed-wide life cycle cumulative energy demand reduction are provided for potential cumulative energy savings (Equation 8):

(8) $$C_{s}=2.78\times 10^{5}\times P_{e}\times E_{s}$$

where,

*C*_*s*_ = potential energy cost savings in $

*E*_*s*_ = cumulative energy demand savings (TJ) (obtained from Equation 2)

*P*_*e*_ = energy price, ($0.103/kWh) (USEIA 2014)

2.78 × 10^5^ = a conversion factor (i.e., 1TJ = 2.78 × 10^5^ kWh)

The cumulative energy savings (*E*_*s*_) for domestic RWH and agricultural RWH systems adopted to watershed-scale were calculated using Equation 2, and corresponding potential energy cost savings *C*_*s*_ were estimated by Equation 8.

Combined with life cycle costs, LCIA impact reduction provides a more comprehensive perspective on economic viability of agricultural RWH systems. We discuss the economic viability of RWH by combining the initial capital cost of agricultural RWH system with the cumulative life cycle energy savings (see Supplementary Material for LCCA details). The economic viability of agricultural RWH involves combining the cumulative energy cost savings with life cycle costs of installing, replacing, operating and maintaining a near-optimal agricultural RWH system. The contributions of cumulative energy cost savings to recouping RWH system life cycle costs are discussed by comparing them to conventional irrigation water prices. Economic viability of domestic RWH was not included because it is not a primary consideration for its adoption.

We also performed sensitivity analysis of future discount rates to LCCA, from 3% to 10%, similar to the Department of Energy’s minimum and maximum real discount rates, as stated by the Code of Federal Regulations 10 CFR 436, Federal Energy Management and Planning Programs (§ 436.14, page 521) (Register 1999).

## Results and discussion

Compared to conventional water supply watershed-wide domestic RWH adoption in the three watersheds reduced the 14 LCIA impact scores, except for ecotoxicity impact of domestic RWH (Table 2). The exception in ecotoxicity impact was consistent with the greater functional unit (per household) ecotoxicity score at 7.3 × 10^−04^ Comparative Toxic Units/m^3^ (CTU/m^3^) for near-optimal domestic RWH than that of the conventional municipal water supply at 6.3 × 10^−04^ CTU/m^3^ as reported by Ghimire et al. (2014). The ecotoxicity factor CTU is an estimate of the potentially affected fraction of species integrated over time and volume, per unit mass of a chemical emitted. The characterization factor for human toxicity impacts is the estimated increase in morbidity in the total human population, per unit mass of a chemical emitted, assuming equal weighting between cancer and non-cancer.

Agricultural RWH adoption in the three watersheds reduced all 14 LCIA impact scores compared to conventional well water irrigation (Table 3). Impact reductions varied with RWH adoption rates and the watershed in consideration: domestic RWH impact reductions for Sycamore and Greens Mill were twice those of Back Creek, and reductions due to agricultural RWH were 17 times higher for Back Creek than Sycamore. Importantly, savings from agricultural RWH for life cycle energy demand were 10 times greater than domestic RWH in Back Creek watershed, but lower (~0.4 times domestic RWH savings) in Sycamore and Greens Mill, offering the opportunity to provide economic incentives (e.g., tax incentives) for agricultural RWH adoption in a watershed with a greater number of farms (Tables 2 and 3).

For 25% domestic RWH adoption rate, impact reductions ranged from 21–43 TJ cumulative energy demand, 267–536 Mg oil eq fossil fuel depletion, 1167–2343 Mg CO_2_ eq. global warming potential, 15–31 Mkg H+ mole eq. acidification, 76–153 Mkg O_3_ eq. smog, 3–6 Mm^3^ blue water use, 10–20 Mkg N eq. eutrophication, and 1–2 Mkg PM_2.5_ eq. human health criteria pollutants (Table 2). With a 25% adoption of agricultural RWH, the impact reductions ranged from 12–210 TJ cumulative energy demand, 594–10092 Mg CO_2_ eq. global warming potential, and 39–2 Mm^3^ blue water use (Table 3). Reductions were higher in Back Creek for agricultural RWH than domestic (from 4 – 41 times) but lower in the other two watersheds (from 10% − 60% of domestic RWH reductions), except for metal depletion, ecotoxicity, and human health-cancer impacts (Tables 2 and 3). The reduction in other environmental dimensions such as ecotoxicity impact due to 100% agricultural RWH watershed-wide over the lifetime of 50 years was substantial at 123602 CTU in Back Creek watershed, although it was negative (at −1069 CTU) due to 100% domestic RWH. Similarly, life cycle reduction in smog impact (as ground level ozone) due to 100% agricultural RWH adoption in Back Creek watershed was 2669 Mkg O_3_ eq., much higher than domestic RWH at 305 Mkg O_3_ eq. Larger agricultural RWH reductions in Back Creek are due to more farms, and larger domestic RWH reductions in Greens Mill watershed are due to more households. The reduction in watershed scale LCIA score varied by adoption rates, watersheds, and number and type of RWH systems; however, the % reduction differed only by the type of RWH as demonstrated by Equation 2. Percent reduction of watershed-scale domestic RWH impact with respect to conventional municipal water supply ranged from 13% (metal depletion) to 99.9% (blue water use) (Table 2). Similarly, % reduction of watershed-scale agricultural RWH impact with respect to conventional well water irrigation ranged from 20% (metal depletion) to 99.9% (blue water use) (Table 3).

### Differences in life cycle water balance

**Figure 3:**
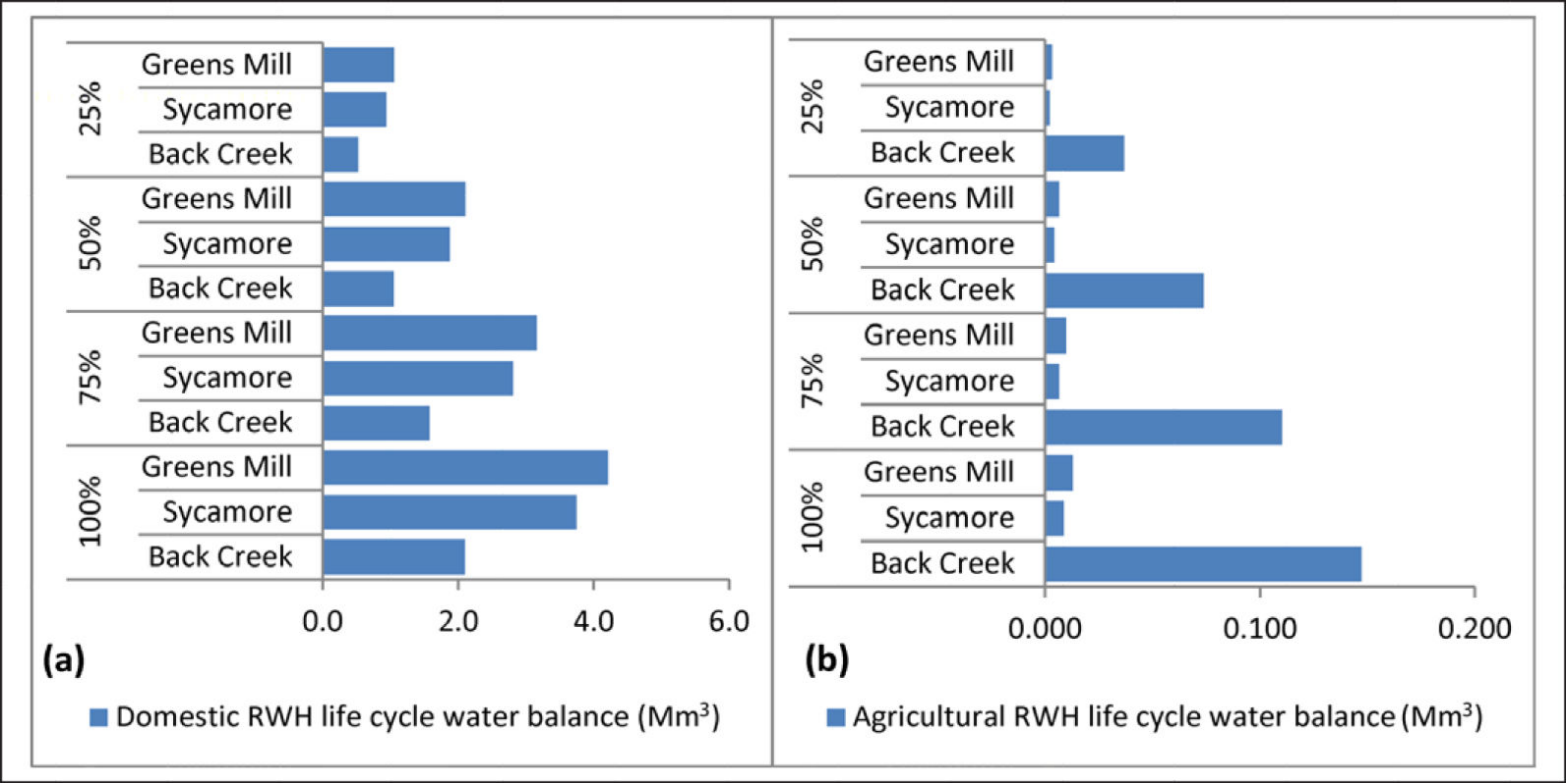
Life cycle water balance for **(a)** domestic RWH adoption of 25%, 50%, 75%, and 100% and **(b)** agricultural RWH adoption of 25%, 50%, 75%, and 100%. DOI: https://doi.org/10.1525/elementa.135.f3

**Figure 4:**
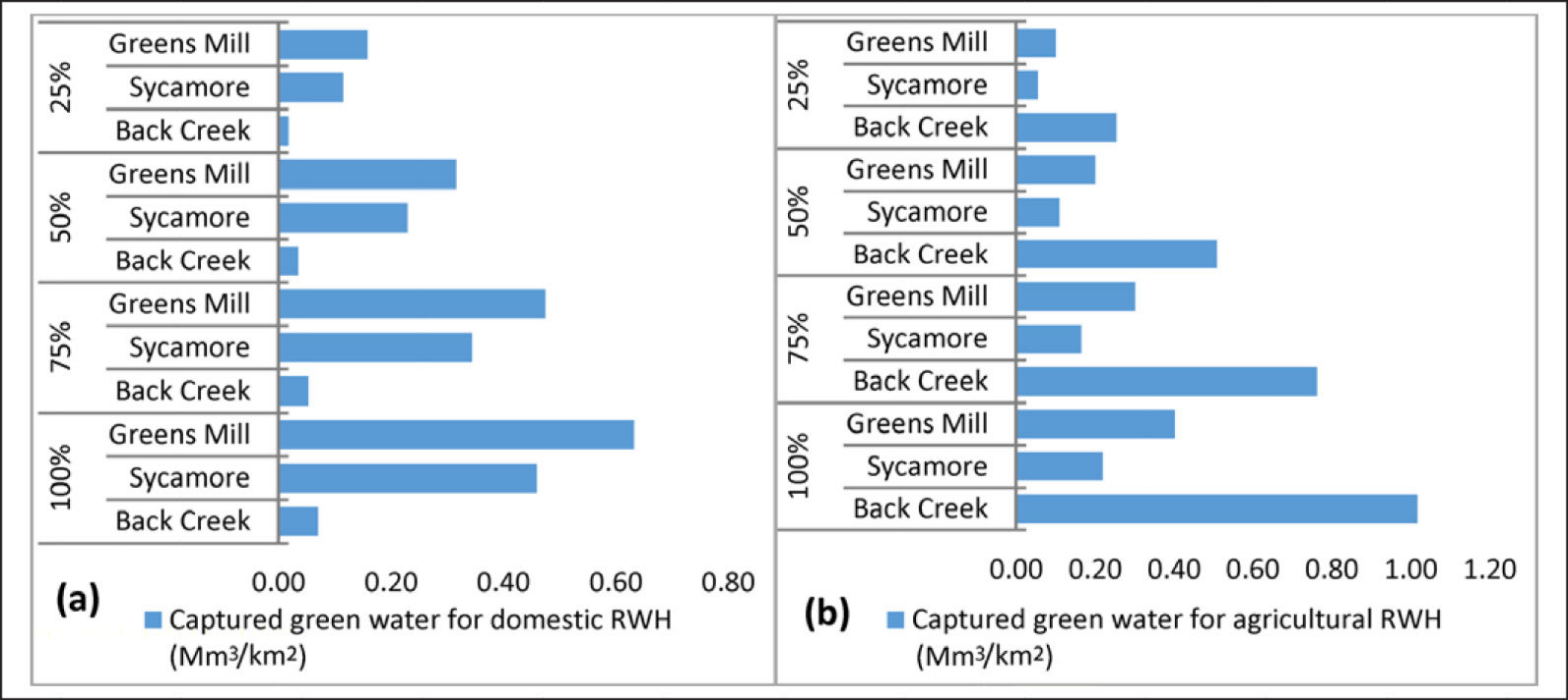
Captured life cycle green water per unit watershed area for **(a)** domestic RWH adoption of 25%, 50%, 75%, and 100% **(b)** agricultural RWH adoption of 25%, 50%, 75%, and 100%. DOI: https://doi.org/10.1525/elementa.135.f4

Agricultural RWH green water use offset blue water use 1:1 (~155 Mm^3^:155 Mm^3^). While agricultural RWH blue water use reductions were 12 times those of the domestic RWH in Back Creek, agricultural RWH reductions were lower (~0.5 times) than domestic RWH reductions in Sycamore and Greens Mill watersheds, due to more households and fewer farms (Tables 2 and 3). Life cycle water balance analysis also showed greater water balance in urban areas with more households and in the rural areas with more farms (Equation 4 and Figures 3 and 4).

Life cycle water balance for 25% domestic RWH adoption ranged from 0.5 – 1.1 Mm^3^ (Figure 3a), suggesting increased surface water availability for downstream ecosystems. This is of particular interest in the light of downstream water availability reduction by 6% due to 25% RWH adoption in Back Creek watershed (Ghimire and Johnston 2013) because from a life cycle point of view, this reduction can be offset by life cycle blue water savings. Also, the water balance analysis for agricultural RWH (Figure 3b) revealed that reducing blue water use approximately equals harvested rainwater, offsetting ground water irrigation. For maximum adoption rates of 100%, the estimated life cycle green water use (or captured rainwater) per unit watershed area for domestic RWH in Greens Mill and agricultural RWH in Back Creek were at 0.63 Mm^3^/km^2^ and 1.02 Mm^3^/km^2^ (Figure 4a, b).

Potential life cycle blue water cost savings from RWH are considerable at larger geographic scales. Considering a domestic water price of $ 1.6/m^3^ (Mayer et al. 1999), potential maximum life cycle cost savings from domestic RWH blue water use reduction (26 Mm^3^) over a lifetime of 50 years are $41.1M for Green Mills watershed. Considering an irrigation water price of $0.1/m^3^ (Wichelns 2010), maximum potential life cycle blue water cost savings from agricultural RWH (reduced blue water use 155 Mm^3^) are $15.5M for Back Creek watershed. Savings increase with rising tiered water price structures, increasing block rates as the water use increases (USEPA 2016). In Atlanta, GA, for example, the average monthly water rate for a family of four using 189.3 liters (50 gallons) per capita per day and 567.8 liters (150 gallons) per capita per day were at $1.76/m^3^ and $2.05/m^3^ in 2015 (Walton 2015), and the life cycle blue water cost savings can be even higher for these water rates. In a 2015 survey of 30 major U.S. cities, the household water bill had increased 41% since 2010 (Walton 2015). The survey also reported that, in 2015, the highest monthly water bill for a family of four using 378.5 liters (100 gallons) per person per day was $153.78/family-month in Santa Fe, New Mexico, equating to $3.39/m^3^, i.e.,

$$\left\lfloor \frac{\$153.78}{month\times 4\frac{persons}{family}\times 30\frac{\mathrm{days}}{\mathrm{month}}\times 100\frac{\mathrm{gallons}}{\mathrm{day}-\mathrm{person}}\times 0.00379\frac{m^{3}}{\mathrm{gallon}}}=\$3.39/{\text{m}}^{3}\right\rfloor$$

and the lowest rate was $ 0.51/m^3^ in Fresno, California.

**Figure 5:**
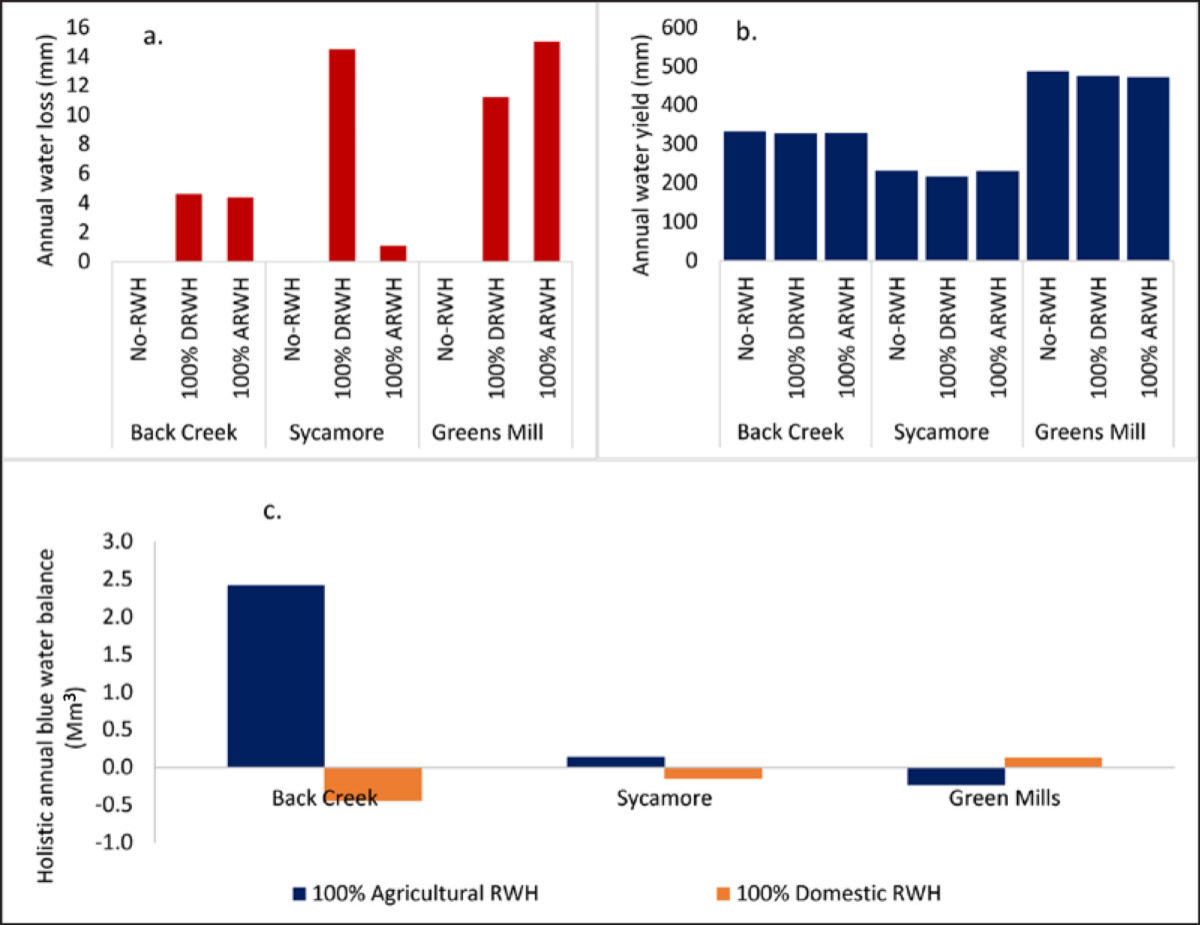
**(a)** annual water loss, **(b)** annual water yield, **(c)** holistic annual water balance for 100% domestic RWH and agricultural RWH adoption in three watersheds. DOI: https://doi.org/10.1525/elementa.135.f5

Note that the life cycle water balance addressed the net savings of embedded blue water by accounting for green water use in all life cycle stages of RWH systems during a service life of 50 years. Further analysis incorporating rainfall effects in life cycle water balance (Equation 5) revealed greater annual holistic blue water savings for 100% agricultural RWH for Back Creek (2.4 Mm^3^/y) and Sycamore Creek (0.1 Mm^3^/y), due to more farms and lower water yield losses. Negative savings were found in Greens Mill due to higher water yield loss and fewer farms (Figure 5a–c). For 100% domestic RWH, holistic blue water use reduction in Greens Mill watershed was higher than others (0.1 Mm^3^/y) due to more domestic RWH systems (Figure 5a). We need to draw an important distinction between LCA impacts and life cycle water impacts. LCA impacts can be combined; however, agricultural and domestic RWH life cycle water balance impacts were modeled separately using the SWAT model. Holistic blue water balance integrating water yields is not additive (100% DRWH and 100% ARWH cannot be combined).

### Differences in cumulative energy cost savings

**Figure 6:**
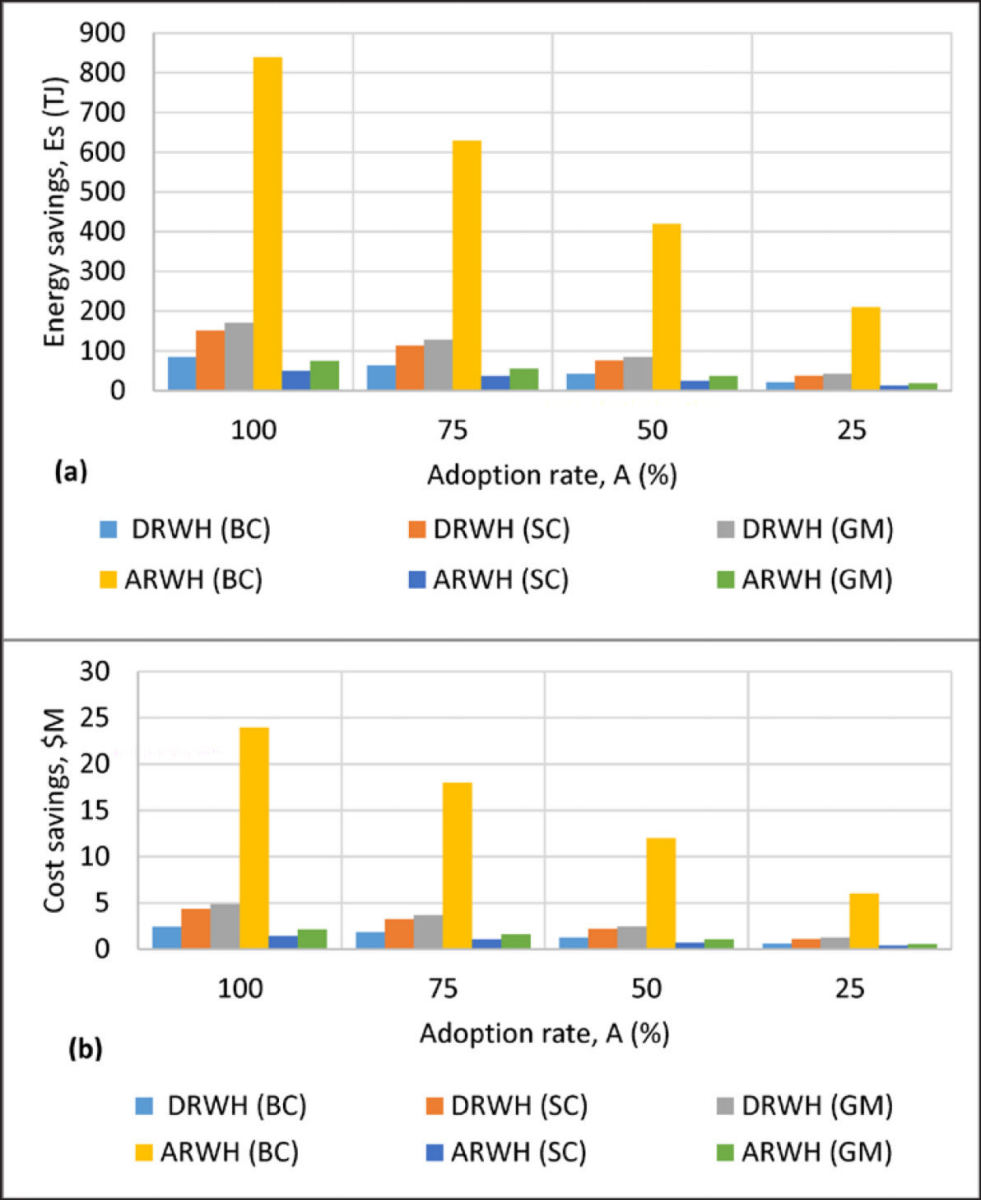
**(a)** Cumulative energy savings and **(b)** life cycle cost savings due to cumulative energy reductions by domestic RWH (DRWH) and agricultural RWH (ARWH) adoption rates in three watersheds: Back Creek (BC), Sycamore Creek (SC), and Greens Mill (GM). DOI: https://doi.org/10.1525/elementa.135.f6

While domestic RWH in Greens Mill realized 57.8% cumulative energy savings when compared to municipal drinking water, agricultural RWH in Back Creek realized 77.7% cumulative energy savings compared to well water irrigation. Cumulative energy savings ranged from 43–171 TJ domestic RWH to 210–838 TJ agricultural RWH savings at adoption rates of 25% and 100% (Figure 6a). Potential energy cost savings (Equation 8) from watershed-wide life cycle cumulative energy use reduction over a lifetime of 50 years are in the millions of dollars. Potential maximum lifetime energy cost savings were estimated at $5M and $24M corresponding to domestic RWH in Greens Mill and agricultural RWH in Back Creek watershedsy (Figure 6b), which are substantial amounts that indicate potential economic viability. This savings is approximately $421/household over the lifetime of domestic RWH systems in Greens Mill. Note that this value does not reflect the potential savings realized by municipalities who may be required to upgrade or replace conventional infrastructure. In Back Creek watershed, $705,515/farm would be realized over the lifetime of agricultural RWH systems. On an annual basis, potential maximum energy coss savings for domestic RWH in Greens Mill was $8.42/household and was $14,110/farm for agricultural RWH in Back Creek.

The economic viability of RWH is further investigated by combining cumulative energy cost savings with initial capital costs and life cycle costs of installing, replacing, operating and maintaining an agricultural RWH system. We estimated initial capital costs of an agricultural RWH system at $271,314 (details in Supplemental Material), equal to $0.060 per m^3^ rainwater supply for irrigation of 4,545,000 m^3^. This price matches approximately the median conventional irrigation water price that ranges from $0.005/m^3^ to $0.10/m^3^ in the U.S. (Wichelns 2010). We estimated life cycle costs of an agricultural RWH system at $0.11/m^3^ (details in Supplemental Material). The life cycle energy cost savings are estimated at $0.16/m^3^ using $6M lifetime savings at the 25% adoption rate of agricultural RWH in Back Creek watershed, i.e., $6M/(0.25 × 34 farms × 4545000 m^3^/farm) (Figure 6b). The difference in estimated life cycle cost of rainwater for agricultural irrigation ($0.11/m^3^) and conventional irrigation ($0.10/m^3^) can be recouped by life cycle cumulative energy cost savings at $0.16/m^3^. LCCA of domestic RWH was not performed because it is not a primary consideration for its adoption.

**Figure 7:**
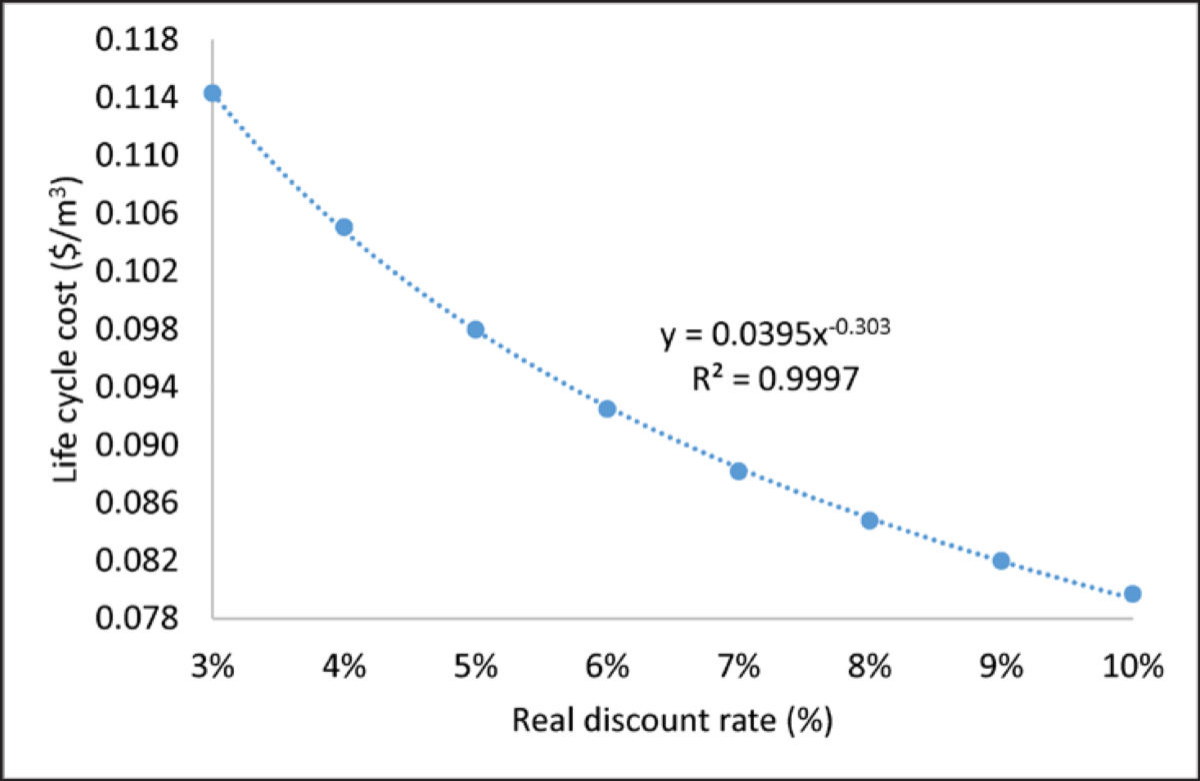
Sensitivity of life cycle costs of agricultural RWH to real discount rates. DOI: https://doi.org/10.1525/elementa.135.f7

The viability of increased RWH adoption is influenced by water requirements, system design, material and energy throughput, and water price, as well as future discount rates (Ghimire et al. 2012). The real discount rate at 3% (*i* = 0.03) was used in all life cycle cost calculations as suggested by the National Institute of Standards and Technology (NIST 2013b). Sensitivity analysis of future real discount rates (3% - 10%) on life cycle costs of agricultural RWH per functional unit of water delivery (1 m^3^) suggested lower RWH life cycle cost with higher discount rates. Agricultural RWH water price ranged from $0.08/m^3^ to $0.11/m^3^, corresponding to 10% and 3% real discount rates (Figure 7). Although cost savings evaluation for remaining impacts and other RWH uses is beyond the scope of this study, environmental and human health impact reductions contribute positively to the overall viability of RWH.

### Energy use and GHG emissions

Energy supplies (coal, natural gas, oil) are the largest source of global GHG emissions (USEPA 2014a), and energy reductions result directly in reduced emission contributions. RWH indicated potential cumulative energy savings as well as reductions in GHG emissions. Total U.S. GHG emissions in 2012 were 6,526 M Metric Tons of CO_2_ eq., with agriculture accounting for approximately 10% (USEPA 2014b). The annual GHG reduction per farm was 24 Metric Tons of CO_2_ eq/Farm-Year (i.e., 40369 Metric Tons of CO_2_ eq/34 farms × 50 y), with substantial reductions possible watershed-wide. Maximum potential GHG emission reduction due to agricultural RWH for the three watersheds varied from 3562–40369 Metric Tons of CO_2_ eq. (Table 3). Life cycle emission reduction through agricultural RWH adoption may have the co-benefit of helping to fulfill U.S. commitments to the United Nations Framework Convention on Climate Change (USEPA 2014b) when considered at the watershed scale.

The methodology is generally applicable to other regions with comparable watershed characteristics, installation costs, and treatment technologies, but it requires appropriate data such as life cycle inventory of used material and energy. Watersheds with higher water yields due to greater rainfall can contribute to water resource sustainability by offsetting water yield losses over life cycle blue water savings of RWH. However, variation in land use/cover type, topography, geology, land surface evaporation, water routing, and quick flow versus impoundments and the social preferences of water sources (e.g., conventional water supplies versus RWH) influence holistic blue water savings but are beyond the scope of this study. We also note that grey and black water (i.e., municipal wastewater) reuse can also increase water resource sustainability, considering that annual coastal wastewater discharge is approximately 6% of the U.S. total estimated water use (NRC 2012). Grey and black water reuse were not considered due to data limitations.

As climate change becomes more severe and communities across the southeastern U.S. experience more frequent droughts (USGCRP 2012), interest in RWH as a climate change adaptation strategy is expected to increase. Attention must be given not only to potential benefits but also to human health and environmental impacts. This study utilized a near-optimal design that minimized material and energy inputs, consistent with Ghimire et al. (2014). Potential energy savings and impact reductions vary with water treatment processes and costs, annual rainfall and water demand, and regulatory standards and design requirements. Tradeoffs may also exist between other irrigation water sources such as rivers versus well water, primarily due to pumping energy caused by spatial variation from pumping distance and necessary pump head.

## Conclusions

Societies across the U.S. and around the world require reliable water supply in the face of population growth and climate change. RWH is being recognized not only as a viable decentralized water supply option for augmenting modern centralized water infrastructures that are costly and resource intensive but also as a green infrastructure strategy for climate change adaptation. Policies are changing to encourage RWH adoption, but RWH sustainability (including unintended environmental consequences and economic viability) should be well studied and across scales. We provided a holistic assessment of environmental and economic viability of domestic and agricultural RWH at the watershed scale in the southeastern U.S., evaluating differences in holistic water savings, life cycle energy cost savings, and GHG emission reductions of both RWH systems in addition to life cycle costs of agricultural RWH.

Watershed-scale impact reductions due to agricultural RWH were 17 times higher for Back Creek than Sycamore due to more farms, and domestic RWH impact reductions for Sycamore and Greens Mill were twice those of Back Creek due to more households. Domestic RWH reduced life cycle environmental and human health impacts compared to municipal drinking water, ranging from 57.8% cumulative energy demand to 99.9% blue water use. Agricultural RWH also reduced the environmental and human health impacts compared to well water, ranging from 20% metal depletion to 99.9% blue water use. Life cycle costs combined with impact reductions, and benefits from shifting to green water over blue water use translates directly to water resource sustainability in terms of return on investment and blue water availability by offsetting surface and ground water consumption. While the difference in estimated life cycle cost of rainwater for agricultural irrigation versus conventional irrigation cost can be recouped by life cycle cumulative energy savings at $0.16/m^3^, the holistic water balance analysis revealed that reducing blue water use approximately equals harvested rainwater. Increased green water use over blue water, reduced energy demand, savings in life cycle energy costs, and decreased global warming potential indicate the potential of RWH as a sustainable water resource management strategy.

Policies encouraging RWH practices, allowing, defining, and clarifying RWH use for various purposes due to climate change are increasing across the U.S.oin Texas, Ohio, Virginia, North Carolina, Illinois, and California, and internationally to Australia, the U.K., Canada, and India (TWDB 2006; GANC 2009; EA 2010; VDH 2011; NCSL 2014). While many states allow rainwater for non-potable purposes, states such as Ohio and Texas allow the practice for potable purposes as well (NCSL 2016). This study more comprehensively evaluates potential economic and environmental benefits of RWH at a range of adoption rates, 25%, 50%, 75%, and 100% and identifies potential economic savings. RWH adoption, even at the lower end of the spectrum, has positive implications for water balance, energy savings, and GHG emission reductions. This systems approach to impact assessment also avoids unanticipated effects of cumulative impacts between different product systems (e.g., RWH components) which can lead to unintended consequences.

There are limits to our study related to inherent limitations of LCA Attribution of ecological and human health impacts to a specific geographic scale, i.e., local versus national or regional, is a known limitation of current LCA methodology. As an example, eutrophication, although considered regional, may or may not be realized within the assessment area boundary due to the use of surrogate data from Ecoinvent. This study builds on the previous LCI database (Ghimire et al. 2014), Building for Environmental and Economic Sustainability (BEES) (NIST 2013a), U.S. LCI (NREL 2013), and a European database (Ecoinvent 2012). Although use of surrogate data in the life cycle inventory is an accepted and necessary practice, the result is that global impacts such as global warming and ozone depletion are generally considered representative of that scale, but local, regional and national impacts must be qualified by the data relied upon. Future research should focus on the data and methods needed to attribute impacts to these various scales of exposure. Other issues such as regulatory requirements on water quality, water withdrawal, and plumbing codes should also be considered. This study intended to inform decentralized RWH decision making for sustainable water management from an increase in heavy downpours to more frequent droughts.

## Supplementary Material

S1

## Figures and Tables

**Table 1:** Characteristics of study watersheds and number of RWH systems with 100% adoption rate, adapted from Ghimire and Johnston (2013). DOI: https://doi.org/10.1525/elementa.135.t1

Watershed	Total area (km^2^)	Average farm area (km^2^)	Total farm area (km^2^)	Number of agricultural RWH systems	Urban area (%)	Number of domestic RWH systems
Back Creek	152	0.34	11.7	34	18	5,768
Sycamore	41.5	0.42	0.91	2	49	10,296
Greens Mill	33.9	1.6	4.6	3	62	11,582

**Table 2:** Reduction in watershed scale life cycle impacts of domestic RWH adoption, with respect to conventional municipal drinking water, in three watersheds: Back Creek = BC, Sycamore Creek = SC, and Greens Mill = GM. CTU = Comparative Toxic Units. DOI: https://doi.org/10.1525/elementa.135.t2

		Reduction in watershed-scale impacts of domestic RWH adoption in three watersheds												% Reduction of impacts
		100%			75%			50%			25%			
LCA impact category	Unit	BC	SC	GM	BC	SC	GM	BC	SC	GM	BC	SC	GM	
Energy Demand	TJ	85	152	171	64	114	128	42	76	85	21	38	43	57.8
Fossil Fuel Depletion	Mg oil eq	1068	1906	2144	801	1429	1608	534	953	1072	267	476	536	42.8
Global Warming	Mg CO_2_ eq	4667	8331	9372	3501	6248	7029	2334	4166	4686	1167	2083	2343	51.7
Metal Depletion	Mg Fe eq	50	89	100	37	66	75	25	44	50	12	22	25	13.2
Ozone Depletion	Mg CFC11 eq	0.0004	0.0008	0.0008	0.0003	0.0006	0.0006	0.0002	0.0004	0.0004	0.0001	0.0002	0.0002	81.8
Acidification	Mkg H+ mole eq	62	no	124	46	83	93	31	55	62	15	28	31	90.1
Smog	Mkg O_3_ eq	305	544	612	229	408	459	152	272	306	76	136	153	61.3
Green Water Use	Mm^3^	−11	−19	−21	−8	−14	−16	−5	−10	−11	−3	−5	−5	--
Blue Water Use	Mm^3^	13	23	26	10	17	19	6	11	13	3	6	6	99.9
Ecotoxicity	CTU	−1069	−1908	−2146	−802	−1431	−1610	−534	−954	−1073	−267	−477	−537	−15.9
Eutrophication	Mkg N eq	39	70	79	30	53	59	20	35	40	10	18	20	90.3
Human Health Criteria	Mkg PM2.5 eq	4	7	8	3	6	6	2	4	4	1	2	2	88.0
Human Health, Cancer	CTU	0.00004	0.00008	0.00009	0.00003	0.00006	0.00007	0.00002	0.00004	0.00004	0.00001	0.00002	0.00002	13.9
Human Health, NonCancer	CTU	0.0005	0.0010	0.0011	0.0004	0.0007	0.0008	0.0003	0.0005	0.0005	0.0001	0.0002	0.0003	84.6

**Table 3:** Reduction in watershed scale life cycle impacts of agricultural RWH adoption, with respect to conventional well water, in three watersheds: Back Creek = BC, Sycamore Creek = SC, and Greens Mill = GM. CTU = Comparative Toxic Units. DOI: https://doi.org/10.1525/elementa.135.t3

		Reduction in watershed-scale impacts of agricultural RWH adoption in three watersheds												% Reduction of impacts
		100%			75%			50%			25%			
LCA impact category	Unit	BC	SC	GM	BC	SC	GM	BC	SC	GM	BC	SC	GM	
Energy Demand	TJ	838	49	74	629	37	55	419	25	37	210	12	18	77.7
Fossil Fuel Depletion	Mg oil eq	15032	884	1326	11274	663	995	7516	442	663	3758	221	332	77.6
Global Warming	Mg CO_2_ eq	40369	2375	3562	30277	1781	2671	20185	1187	1781	10092	594	890	75.8
Metal Depletion	Mg Fe eq	2018	119	178	1514	89	134	1009	59	89	505	30	45	20.1
Ozone Depletion	Mg CFC11 eq	0.00210	0.00012	0.00019	0.00158	0.00009	0.00014	0.00105	0.00006	0.00009	0.00053	0.00003	0.00005	67.2
Acidification	Mkg H+ mole eq	265	16	23	199	12	18	133	8	12	66	4	6	86.7
Smog	Mkg O_3_ eq	2669	157	236	2002	118	177	1335	79	118	667	39	59	79.5
Green Water Use	Mm^3^	−154.5	−9	−14	−116	−7	−10	−77	−5	−7	−39	−2	−3	---
Blue Water Use	Mm^3^	154.7	9	14	116	7	10	77	5	7	39	2	3	99.9
Ecotoxicity	CTU	123602	7271	10906	92702	5453	8180	61801	3635	5453	30901	1818	2727	71.8
Eutrophication, total	Mkg N eq	147	9	13	111	7	10	74	4	7	37	2	3	76.9
Human Health Criteria	Mkg PM2.5 eq	19.2	1.1	1.7	14.4	0.8	1.3	9.6	0.6	0.8	4.8	0.3	0.4	66.8
Human Health, Cancer	CTU	0.00142	0.00008	0.00013	0.00107	0.00006	0.00009	0.00071	0.00004	0.00006	0.00036	0.00002	0.00003	58.3
Human Health, NonCancer	CTU	0.0062	0.0004	0.0005	0.0047	0.0003	0.0004	0.0031	0.0002	0.0003	0.0016	0.0001	0.0001	62.3

## Abbreviations

Acronym: Description
BC: Back Creek
BU: Blue water use
CFR: Code of Federal Regulations
CTU: Comparative toxic units
EPA: U.S. Environmental Protection Agency
GHG: Greenhouse gas
GM: Greens Mill
GU: Green water use
HUC: Hydrologic Unit Code
LCA: Life cycle assessment
LCCA: Life cycle cost assessment
LCI: Life cycle inventory
LCIA: Life cycle impact assessment
MDW: Municipal drinking water
NIST: National Institute of Standards and Technology
RWH: Rainwater harvesting
SC: Sycamore Creek
SM: Supplemental Material
SWAT: Soil and Water Assessment Tool
TRACI: Tool for the Reduction and Assessment of Chemical and Other Environmental Impacts
WW: Well water
