# Supplementary material for: Holistic impact assessment and cost savings of rainwater harvesting at the watershed scale
Source: Elementa (Wash D C). Author manuscript; Available in PMC 2019 Jul 18. (PMC6638562; doi:10.1525/elementa.135)
Supplement: S1 [file NIHMS985940-supplement-S1.pdf]

SM 2. Description of Costs

We computed the life cycle cost of an agricultural RWH system by estimating initial investment, as well as the present value of future costs of components incurred during the service life (Table S1). All initial investment costs occurring in the base date (Year 2014) were not discounted since they were in present value. Future costs of component replacements occurring at the end of service, one-time, and at non-annual intervals were discounted from the end of service life to calculate present value. Residual values of the capital investments were estimated using straight-line depreciation. For annually recurring costs such as operation and maintenance, we considered real discounting rate addressing the time value of money. Below, we describe the computation procedures of life cycle costs (summarized in Table S1) consistent with engineering economics (Revelle et al. 2004) and life cycle cost analysis guidelines of the National Institute of Standards and Technology (Fuller and Petersen 1996). We describe initial capital investments costs (e.g., costs of sediment chamber (pond), pipe, pivot center, and valves); replacement costs (e.g., pivot center replacement at the end of 20 and 40 years); residual value; and costs that recur annually such as operation and maintenance (O&M) and sediment dredging and disposal.

Initial Capital Investment Costs

Sediment Chamber (Pond) Cost

The cost of a sediment chamber (pond) is calculated based on cost per unit volume, V_p (\$3.8/m^3) of a pond, as suggested by the Virginia FY14 Environmental Quality Incentive Program (EQIP) Payment Schedule (USDA 2014) (Equation S1).

$$C_{pond} = 3.8 \times V_p \qquad [S1]$$

Polyethylene Tank Cost

The cost of polyethylene tank (C_{tank}) ($) is based on tank volume, V_t (m^3), as provided by the State of Michigan (SOM 2003):

$$C_{tank} = 297.64 \text{ x } V_t + 161.68 \quad [S2]$$

Pivot Center Cost

The cost of a typical pivot center, C_{pivot} ($/ha), is derived from cost per unit of irrigated farm area, a (Bliesner and Spare 2001):

$$C_{pivot} = 8215.1 \text{ x } a^{-0.565} \quad [S3]$$

Replacement Cost

Replacement costs are one-time costs at non-annual intervals. Replacement occurred at the end of service life of a component: pivot center and valves were replaced at 20 and 7.5 years, respectively. The present value of each replacement cost (R_{PV}), *$,* was estimated using the corresponding single present value (SPV) discount factor:

$$R_{PV} = F_t \text{ x } \left(1+i\right)^{t} \quad [S4]$$

where,

$(1+i)^{-t}$ = SPV discount factor, with t being the service life of a component of agricultural RWH system (20 years for pivot center and 7.5 years for check valve)

i = the real discount rate (0.03) suggested by the National Institute of Standards and Technology (NIST 2013). We also performed sensitivity analysis of i from 3% to 10% (Register 1999).

F_t = Future price of an item such as agricultural RWH pivot center, \$, estimated as the replacement cost at replacement times (multiple replacements of the component's service life less than or equal to 50). We estimated future price, F_t, as identical to the base date cost, assuming zero real price escalation rates (the rate of price change ≅ general price inflation rate), as suggested by the NIST guideline (Fuller and Petersen 1996).

Using Equation S4, we estimated present value for every replacement in the future and summed them to reach the component's total present value. For example, replacement costs of two units of pivot center at the end of service life of 20 and 40 years is the base date cost at $38,296 each (Table S1). The present value of total future replacement costs of two pivot centers with the base date (2014) unit price of $38,296 was therefore estimated as:

$$R_{PV} = 38{,}296 \text{ x } (1+.03)^{-20} + 38{,}296 \text{ x } (1+.03)^{-40} = \$\ 32{,}943 \qquad [S5]$$

We note that actual future replacement cost of an item, with a price escalation rate deviating from general inflation rates, may also be estimated using a nominal price escalation rate such as 3%, as suggested by the NIST life cycle cost assessment handbook; corresponding present value, R_{PV}, of all pivot replacements in an agricultural RWH system may be estimated. For example, in the case of pivot centers, total future costs of two pivot center component replacements may be estimated as:

$$F_t = 38{,}296 \text{ x } (1+.03)^{20} + 38{,}296 \text{ x } (1+.03)^{40} = \$194{,}090 \qquad [S6]$$

And corresponding present value R_{PV} of all pivot replacements in an agricultural RWH system may be estimated at $194,090 x $(1+0.03)^{-50}$ = $44,273, which is greater than the value estimated using Equation S5. We used replacement costs identical to the base date cost, assuming zero real price escalation rates for this analysis.

Residual Value

Residual value at the end of service life of a component was estimated by the straight-line method of depreciation recommended by the Royal Institution of Chartered Surveyors and the U.S. Department of Energy (Fuller and Petersen 1996; RICS 2014). For example, replacement of a pivot center (base date cost, *P*) that operated 10 years of its 20 years of service life would have a residual present value, $V_{PV,}$ of:

$$V_{PV} = \frac{10}{20} \text{ x P } = 0.5 \text{ x } \$\ 38{,}296 = \$19{,}148 \qquad \text{[S7]}$$

The present value of this residual value was then estimated at $4,368, using Equation S4.

Annual Costs

The present value of annual operation and maintenance costs were estimated using the uniform present value (UPV) factor. Water prices vary with location, block price and year, and energy price varies by fuel type, price escalation rate and census region (Fuller and Petersen 1996), but neither were included in our analysis. For all annual costs, we accounted for future discounting by estimating present value (PV) of annually recurring uniform amounts, as defined by:

$$C_{PV} = A \text{ x } \frac{(1+i)^n \quad 1}{i(1+i)^n} \qquad \text{[S8]}$$

where,

C_{PV} = present value of annual cost

A = Annual costs, $

$\frac{(1+i)^n \quad 1}{i(1+i)^n}$ = uniform present value (UPV) factor

i = real discount rate (.03) (NIST 2013)

n = number of compounding years (i.e., service life = 50 years)

We used 1.7% of total investment costs as the O&M costs of an agricultural RWH system (Hogan et al. 2007). Sediment removal is performed once every 2-15 years depending on pond type (Commission 2007), but we estimated costs of annual removal. The volume of sediment dredging was based on 0.02 m depth by 7108 m^2 surface area per year, at \$20/m^3 (Marsalek and Marsalek 1997; USDA 1997; Commission 2007). The surface area assumes average water depth of 1.8 m (6 ft) in a sedimentation chamber of 13000 m^3 volume (Ghimire et al. 2014). Sediment disposal costs were estimated at \$7/m^3 (See Table S1).

REFERENCES

Bare J. 2011. TRACI 2.0: the tool for the reduction and assessment of chemical and other environmental impacts 2.0. *Clean Technologies and Environmental Policy* **13**:687-696.

Bliesner RD, Spare D. Center Pivot Irrigation. In: O'Neill M, editor. Second Annual Four Corners Irrigation Workshop 2001; USA: New Mexico State University. p. 25.

Commission NVR. 2007. Maintaining Stormwater Systems: A Guidebook for Private Owners and Operators in Northern Virginia. Fairfax: Virginia Coastal Zone Management Program at the Department of Environmental Quality.

CPFC. Price Lists [Internet]. USA: Charlotte Pipe and Foundry. Available from: http://www.charlottepipe.com/price_lists.aspx

Flomatic. Flomatic® Valves Price List March 24, 2014 [Internet]. USA: Flomatic Corporation. Available from: http://www.flomatic.com/

Fuller S, Petersen S. 1996. Life-cycle costing manual for the federal energy management program, 1995 Edition. *NIST handbook* **135**.

Ghimire SR, Johnston JM, Ingwersen WW, Hawkins TR. 2014. Life Cycle Assessment of Domestic and Agricultural Rainwater Harvesting Systems. *Environmental Science & Technology* **48**:4069-4077.

Goedkoop MH, R.; Huijbregts, M.; Schryver, A.; Struijs, J.; and Zelm, R. . 2012. ReCiPe 2008 - A life cycle impact assessment method which comprises harmonised category indicators at the midpoint and the endpoint level. Netherlands: Ministry of Housing, Spatial Planning and Environment.

Hischier RAH-JB, C.; Büsser S.; Doka, G.; Frischknecht R.; Kleijer A.; Leuenberger M.; Nemecek, T.; and Simons, A. 2010. Documentation of changes implemented in ecoinvent Data v2.2 Switzerland: Swiss Centre for LCI. No. Final report ecoinvent data v2.2 Dübendorf.

Hogan R, Stiles S, Tacker P, Vories E, Bryant K. 2007. Estimating irrigation costs. Cooperative Extension Service, University of Arkansas, US Department of Agriculture, and county governments cooperating.

Marsalek J, Marsalek P. 1997. Characteristics of sediments from a stormwater management pond. *Water Science and Technology* **36**:117-122.

NIST. 2013. Energy Price Indices and Discount Factors for Life-Cycle Cost Analysis – 2013 Annual Supplement to NIST Handbook 135 and NBS Special Publication 709. National Institute of Standards and Technology, U.S. Department of Commerce.

Register F. 1999. 10 CFR 436 - FEDERAL ENERGY MANAGEMENT AND PLANNING PROGRAMS. In: House TW, editor.: The White House, USA.

Revelle CA, Whitlach EE, Wright JR. 2004. Civil and environmental systems engineering, Second Edition. New Jersey USA: Prentice Hall.

RICS. RICS draft guidance note: Life cycle costing [Internet]. The United Kingdom: Royal Institution of Chartered Surveyors Available from: https://consultations.rics.org/consult.ti/life_cycle_costing/viewCompoundDoc?docid=794580&sessionid=&voteid=&partId=795956

SOM. 2003. Tanks Section UIP 11. USA: State of Michigan.

USDA. 1997. Ponds — Planning, Design, Construction. USA: Natural Resources Conservation Service (NRCS).

USDA. 2014. FY14 EQIP Payment Schedule. USA: United States Department of Agriculture

USEPA. 2012. Tool for the Reduction and Assessment of Chemical and Other Environmental Impacts (TRACI) TRACI version 2.1 User's Guide. Cincinnati, OH, USA: U.S. Environmental Protection Agency. No. EPA/600/R-12/554.

USEPA. Tool for the reduction and assessment of chemical and other environmental impacts (TRACI) [Internet]. USA: U.S. Environmental Protection Agency. Available from: http://www.epa.gov/nrmrl/std/traci/traci.html

WFN. 2009. Water footprint manual Enschede. The Netherlands The Netherlands Water Footprint Network.
